# Janus kinase 1 inhibitors for treating immune checkpoint inhibitor-induced enterocolitis – report of two filgotinib-treated cases and literature review

**DOI:** 10.2340/1651-226X.2025.44298

**Published:** 2025-10-07

**Authors:** Henrik Ekedahl, Gudbjörg Sigurjonsdottir, Viktoria Bergqvist, Björn Båtshake, Ana Carneiro, Jan Marsal

**Affiliations:** a Department of Hematology, Oncology, and Radiation Physics, Skåne University Hospital, Lund, Sweden; b Section of Oncology and Pathology, Department of Clinical Sciences Lund, Lund University, Lund, Sweden; c Department of Gastroenterology, Skåne University Hospital, Lund/Malmö, Sweden; d Section of Medicine, Department of Clinical Sciences Lund, Lund University, Lund, Sweden; e Section of Immunology, Department of Experimental Medical Science, Lund University, Lund, Sweden

**Keywords:** Janus kinase inhibitors, immune checkpoint inhibitors, enterocolitis, colitis, melanoma, case report

## Abstract

**Background and purpose:**

Treatment of cancer with immune checkpoint inhibitors (ICIs) entails a risk of immune-related adverse events (irAEs). Data on the treatment of immune-related enterocolitis (irEC) in patients with inadequate symptom control after treatment with standard therapy including corticosteroids, infliximab, and vedolizumab are scarce. Based on limited data, recommendations include treatment with the pan-Janus kinase (JAK) inhibitor tofacitinib. Filgotinib is a more recently developed JAK inhibitor with preferential inhibition of JAK1, which might imply a more favorable safety profile. Filgotinib is approved for the treatment of ulcerative colitis and might thus be an option in refractory irEC.

**Patients and methods:**

We present two cases of metastatic melanoma treated with ICIs who developed corticosteroid and infliximab-refractory irEC. Given non-conventional pharmaceutical management, literature review was performed regarding mechanisms of action and safety profiles of JAK inhibitors.

**Results:**

Both patients were treated with filgotinib, which resulted in rapid remission of symptoms in both cases. One of the patients was treated with off-label high-dose filgotinib, which has not been described previously. The rationale and safety regarding the use of JAK1 inhibitors in irAEs are discussed, including the seemingly diverging existing data on potential effects of JAK inhibition on ICI-induced anti-tumoral immune-responses. In addition, the rationale for the high-dose treatment is scrutinized.

**Interpretation:**

This report suggests that filgotinib may be considered for treating irEC refractory to standard therapy.

## Introduction

Immune checkpoint inhibitors (ICIs) are widely used to treat various types of cancers and entail a risk of immune-related adverse events (irAEs), including immune-related enterocolitis (irEC). First-line treatment for irEC is corticosteroids, and in cases of inadequate response or corticosteroid dependency, treatment with the TNFα inhibitor infliximab or the integrin inhibitor vedolizumab is recommended [1–3]. In patients with irEC refractory to standard treatment, American Society of Clinical Oncology (ASCO) and European Society For Medical Oncology (ESMO) guidelines recommend considering fecal microbiota transplant, the Janus kinase inhibitor (JAKi) tofacitinib, or the IL-12/23-blocking antibody ustekinumab, based on very small sample sizes [1, 2].

JAKis constitute an emerging alternative for the treatment of several immune-mediated inflammatory diseases (IMIDs), including inflammatory bowel disease (IBD) [4]. JAKis are orally administered small molecule drugs with quick absorption and short half-life [5]. The onset of action is rapid with reports of significant improvements of symptoms within 3 days [6, 7]. Tofacitinib, a pan-JAKi with preferential inhibition of JAK1 and JAK3, was the first JAKi to gain regulatory approval for the treatment of ulcerative colitis (UC) in 2018 [8]. Case reports describe symptom remission after tofacitinib treatment of irEC refractory to standard treatment [9–14]. Furthermore, continued therapeutic responses to ICIs have been described after tofacitinib treatment [10, 12, 13]. However, increased rates of major adverse cardiovascular events (MACEs), serious infections, venous thromboembolism (VTE), cancer, and death were described for rheumatoid arthritis (RA) patients treated with tofacitinib in a post-registration safety trial [15], which led the U.S. Food and Drug Administration (FDA) and European Medicines Agency (EMA) to issue warnings for all approved JAKis [16, 17]. However, this has caused debate, since other studies have not confirmed the reported increased risks [18–21].

Filgotinib is a more recently developed JAKi with preferential selectivity for JAK1 (one of four known JAKs), which might imply an improved safety profile [22–24]. Filgotinib is approved for the treatment of patients with UC [25]. To our knowledge, there have been no previously reported cases of irEC treated with filgotinib nor any previously published cases of any type treated with off-label high-dose filgotinib. Here, we present two cases of steroid-refractory irEC experiencing rapid responses to filgotinib, one of which received off-label high-dose filgotinib.

## Case description

### Case 1

A 77-year-old female with no significant comorbidities was diagnosed with liver metastases of choroidal melanoma. She was asymptomatic, and routine blood tests were normal. In October 2023, the patient was initiated on ICI therapy with ipilimumab 3 mg/kg and nivolumab 1 mg/kg (ipi/nivo) administered Q3W. After the third ipi/nivo cycle, the patient was diagnosed with grade 2 ir-hepatotoxicity. She was successfully treated with prednisolone 1 mg/kg, tapered over 5 weeks. A CT scan performed after three cycles of ipi/nivo revealed unconfirmed progressive disease (UPD) in the liver.

**Figure 1:**
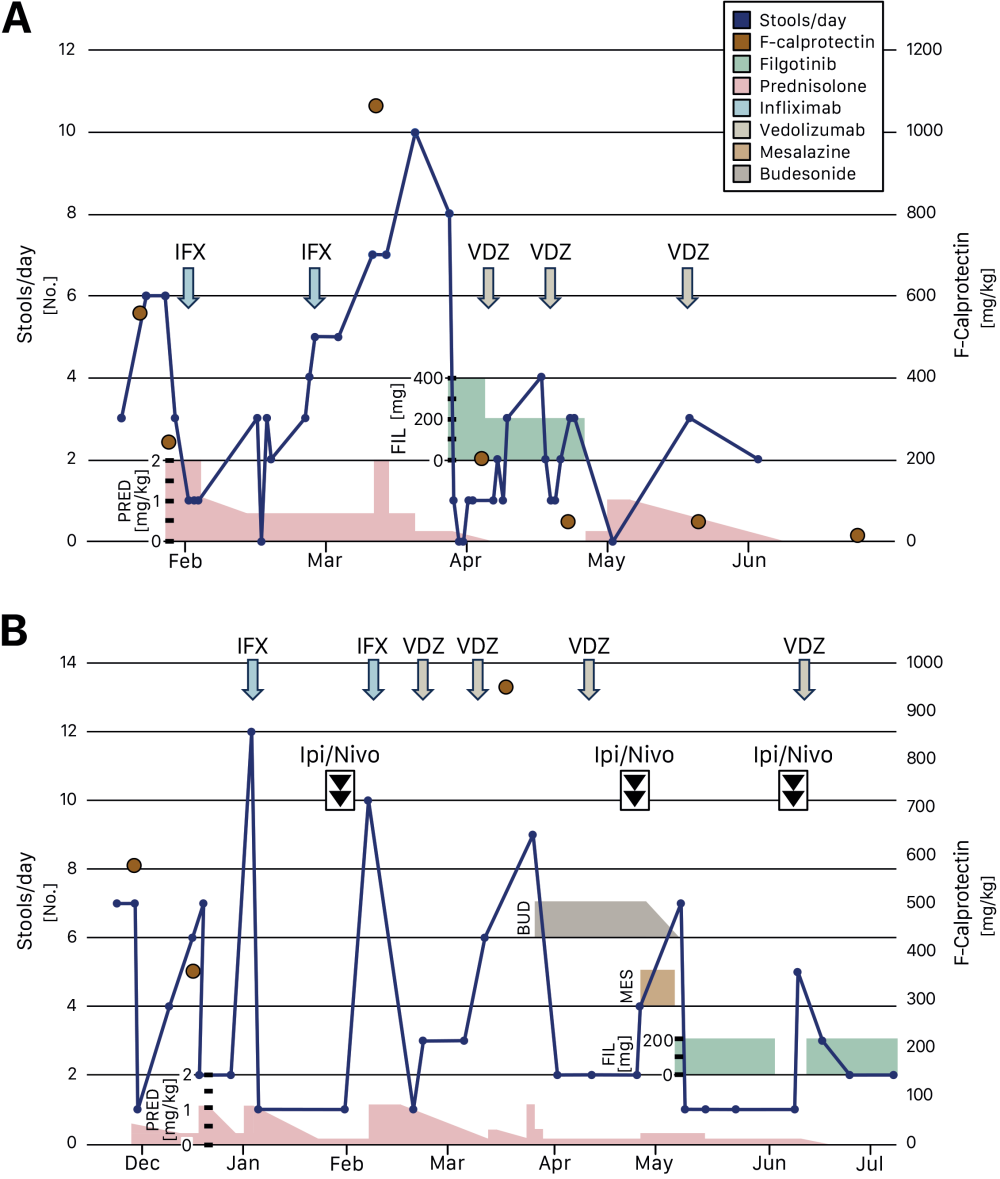
The clinical course of case 1 (A) and case 2 (B). Stool frequency and levels of fecal calprotectin depicted in relation to given treatments. BUD: budesonid (max dose 9 mg QD); IFX: infliximab 5 mg/kg; Ipi/Nivo: ipilimumab+nivolumab; F: fecal; FIL: filgotinib; MES: mesalazine 5 g QD; No: number; PRED: prednisolone; QD: once daily; VDZ: vedolizumab 300 mg.

Shortly after the fourth cycle, the patient reported grade 1 diarrhea. Work-up (fecal cultures and *C. difficile* toxin test) did not support any infectious cause, but fecal calprotectin was elevated (554 mg/kg, reference: <80 mg/kg), suggesting an inflammatory cause. The diarrhea progressed with the onset of fever. An abdominal CT scan showed moderate edema of the rectosigmoidal wall and low-grade edema in the transverse and descending colon. Under the diagnosis of grade 3 irEC, the patient was started on methylprednisolone 2 mg/kg intravenously (IV) and IV antibiotics with only minor improvement. After 4 days, one dose of infliximab 5 mg/kg was given. Within 3 days, the patient reported normalized stool frequency and consistency, and prednisolone tapering was initiated (Figure 1A).

Two weeks after the infliximab administration, the patient reported recurrence of diarrhea. A second dose of infliximab was administered along with an increase of the prednisolone dose to 2 mg/kg; however, there was no improvement of symptoms. A new CT scan showed edema of the colonic wall. The prednisolone dose was lowered, and a repeated work-up was performed. All tests for an infectious cause, including cytomegalovirus (CMV), were negative. The patient’s clinical status was quickly deteriorating, necessitating prompt intervention to relieve the inflammation. The patient was started on filgotinib 400 mg per day (200 mg BID). Filgotinib at this high dose was chosen over vedolizumab, which has a slower onset, to optimize the likelihood of achieving remission in the face of irEC grade 3 resistant to corticosteroids and infliximab. The patient experienced a rapid improvement with normalized stool frequency and consistency within 1 day. After 1 week of treatment, the patient remained symptom-free, with a corroborative decrease in fecal calprotectin (Figure 1A). The filgotinib dose was lowered to 200 mg QD, prednisolone was discontinued, and vedolizumab was started.

Due to confirmed progression of liver metastases, ipi/nivo was discontinued, and the patient received selective internal radiation therapy (SIRT) of the liver in April 2024. Filgotinib was discontinued the day before the procedure, after 4 weeks of treatment. The day after SIRT was performed, the patient experienced severe vertigo. She was diagnosed with vestibular neuritis and started methylprednisolone 1 mg/kg IV for 5 days with a gradual, albeit not complete, improvement of the vertigo. Vedolizumab was discontinued after four doses since the patient had no diarrhea and normal fecal calprotectin levels.

### Case 2

A 74-year-old male without significant comorbidities presented with hepatic and mesenterial metastases of cutaneous melanoma, elevated lactate dehydrogenase (LDH) levels (>2 x ULN), weight loss, and abdominal discomfort.

Nine days after the initiation of ipi/nivo, the patient reported grade 3 diarrhea. A CT scan did not show signs of enterocolitis, the infection work-up was negative, but fecal calprotectin was elevated to 577 mg/kg. Prednisolone (0.5 mg/kg) was started for suspected irEC, resulting in rapid remission of symptoms (Figure 1B). The diarrhea did however recur twice during prednisolone tapering. Therefore, the patient received one dose of infliximab 5 mg/kg, resulting in immediate complete remission of symptoms, and prednisolone was tapered successfully.

Grade 3 diarrhea recurred after the second cycle of ipi/nivo. This time, prednisolone 1 mg/kg and one dose of infliximab 5 mg/kg resulted in only a partial clinical response. Hence, vedolizumab treatment was started. The patient continued to report mild diarrhea, which prompted further investigation with a colonoscopy. This showed only a mild enhancement in vascularity and erythema, and biopsies showed discrete superficial mucosal inflammatory activity without signs of CMV infection. Under the suspicion of ir-microscopic enterocolitis, oral budesonide 9 mg QD was started in addition to vedolizumab. Symptoms improved, and prednisolone was gradually tapered off.

The third cycle of ipi/nivo was given with flipped dosing (ipilimumab 1 mg/kg and nivolumab 3 mg/kg), with the patient still on budesonide 9 mg QD. The following day, the patient reported recurrence of grade 3 diarrhea. Mesalazine 5 g QD was started, but no improvement was observed. After 2 weeks, the patient reported vomiting, abdominal pain, and grade 3 diarrhea. The patient’s condition was judged to once again represent grade 3 irEC, and the patient was started on filgotinib 200 mg QD. He reported clinical improvement within 1 day and complete remission of symptoms within 5 days (Figure 1B).

Filgotinib was discontinued after 4 weeks of treatment. Five days later, the fourth cycle of ipi/nivo (flipped dosing) was administered. Again, grade 3 diarrhea recurred after 2 days. Filgotinib was re-started with gradual improvement of symptoms and complete remission after 4 weeks. A CT scan showed progressive neoplastic disease, and second-line treatment with BRAF and MEK inhibitors dabrafenib and trametinib was initiated. Filgotinib treatment was discontinued after an additional 4 weeks of treatment. Two months later, the patient was still free of irEC symptoms.

## Discussion

The rationale for using JAKis in the treatment of irAEs is based on a recognized role of cytokines in irAE, the characterization of JAKs as key regulators of lymphocytes, the associations between several IMIDs and JAK polymorphisms (possibly causing increased interferon responses), and the proven efficacy of JAKis in the treatment of IBD [4].

Due to lack of response to high-dose prednisolone and infliximab, with quickly deteriorating clinical status, the patient in case 1 initially received treatment with filgotinib at an off-label high-dose, with a planned reduction to standard dosing after 1 week. Although vedolizumab occasionally may entail relatively rapid symptom control, cases with infliximab-refractory irEC often require longer treatment before a significant improvement is observed [26]. Therefore, we utilized filgotinib to enhance the chances of reaching remission with the subsequent application of vedolizumab, which, given its GI-selective immunosuppressive effect, is more suitable for maintenance therapy. To our knowledge, there have not been any previous reports on off-label high-dose filgotinib used in irEC or any other indication.

The high-dose regimen is supported by studies on tofacitinib and upadacitinib in UC [27–29]. Furthermore, a pharmacodynamic study suggests that filgotinib 200 mg QD is equivalent to tofacitinib 5 mg BID and upadacitinib 15 mg QD, which are clearly suboptimal induction doses in IBD [25, 30–32]. In addition, the dose-finding studies for filgotinib were evaluated using peripheral whole blood (which likely does not reflect the conditions in an edematous bowel wall) and were designed for the treatment of rheumatoid arthritis (RA), which consistently requires lower drug-doses compared with IBD [25, 33–35].

Although there are no prospective comparative safety studies on JAKis, tofactinib treatment is associated with a decrease in lymphocyte counts, which is not the case with the preferential JAK1 inhibitors filgotinib and upadacitinib [36]. In a meta-analysis, the relative risk for herpes zoster was higher for patients who were treated with tofacitinib compared with filgotinib or upadacitinib [18]. In case 1, the patient was diagnosed with vestibular neuritis. Although viral infections, including herpes zoster, have been suggested as an important cause of vestibular neuritis [37], vestibular neuritis has not been reported as an adverse effect of JAKis. The ORAL Surveillance trial reported increased risks of MACE, serious infections, VTE, and cancer for tofacitinib therapy compared with TNF-inhibitors in patients with RA aged >50 years who had ≥1 cardiovascular risk factor [15]. However, the results have not been corroborated by other studies [18–21, 38]. Thus, the evidence is ambiguous, and the risks should be weighed against the risks of undertreating irAEs or the cancer.

The JAK-STAT pathway plays crucial roles in cancer, including regulating immune surveillance [39]. Interferon-γ, which activates JAK1 and JAK2, can exert direct antiproliferative effects on tumor cells, upregulate expression of antigen-presenting major histocompatibility complex (MHC) molecules, and inhibit immune suppressive regulatory CD4^+^ T-cells (T_regs_) [40]. Inactivating mutations in JAK1 and JAK2 have been described as a cause of acquired resistance to PD-1 inhibitor treatment due to a lack of response to interferon-γ-induced growth arrest [41]. Furthermore, JAK1/2 mutations have been shown to cause primary resistance to PD-1 inhibitors, associated with a lack of T-cell infiltrates [42]. Loss-of-function JAK1/2 alterations were associated with a shorter overall survival in the Cancer Genome Atlas Program (TCGA) Skin Cutaneous Melanoma dataset [42]. On the other hand, the JAK/STAT pathway can also exert pro-tumoral effects. Gain-of-function mutations in JAK2 are highly prevalent drivers in myeloproliferative neoplasms and targetable by JAK1/2 inhibitors including ruxolitinib [43]. STAT3 is considered an oncogene, which is often constitutively active in melanoma cell lines [44]. Moreover, interferons can promote adaptive immune resistance by stimulating the expression of PD-L1 [45, 46], and chronic type I interferon signaling promotes terminal exhaustion of CD8^+^ T-cells [45, 47]. Altogether, an early response to interferons may act tumor suppressively, where loss-of-function alterations mediate resistance mechanisms. However, in chronic inflammation, a prolonged interferon exposure might promote PD-L1-dependent and PD-L1-independent immune resistance [45].

Recent studies shed light over the seemingly contradictory roles of JAK inhibition in cancer. In melanoma cells with malfunctioning interferon-γ signaling, there is an upregulation of the mTOR pathway, resulting in JAK1/2 activation [48]. Treatment with ruxolitinib resulted in improved effector functions of CD8^+^ T-cells, reduced frequency of intratumoral T_regs_, and suppression of melanoma cells. In accordance with these findings, two studies recently showed that JAKis increased the anti-tumoral effects of ICIs [49, 50]. Zak et al. reported that the inhibition of JAK1 and/or JAK2 increased the frequency of CD8^+^ T-cells, and that ICI in combination with ruxolitinib treatment reduced tumor growth more effectively than ICI alone in lymphoma and lung cancer cell models [49]. In a phase I trial, 21 patients with Hodgkin lymphoma, who had previously failed ICI therapy, were treated with ruxolitinib and from day 8 also with nivolumab. There was no dose-limiting toxicity, and 10 out of 19 (53%) evaluable patients showed objective responses with a 2-year progression-free survival of 46% [49]. Mathew et al. showed that the JAK1 inhibitor itacitinib improved ICI efficacy in CTLA-4 inhibitor-resistant melanoma in mice through antagonizing interferon type I signaling [50]. Subsequently, 21 patients with treatment-naïve metastatic non-small cell lung cancer, with tumor PD-L1 ≥ 50%, were treated with pembrolizumab Q3W and itacitinib during pembrolizumab cycles 3 and 4. The best overall response rate was 67%, and the median progression-free survival was nearly 2 years [50]. Taken together, interferon signaling through the JAK/STAT pathway may have both tumor suppressing and stimulating effects depending on timing and genetic context, but recent studies suggest that JAKis might enhance the therapeutic effects of ICIs.

## Conclusion

Filgotinib, a preferential JAK1 inhibitor, might be used to induce remission of irEC in patients who have received standard treatment with corticosteroids and infliximab. One of the cases herein showed that it is feasible to use high-dose filgotinib, for which other studies have provided sound theoretical rationale although it has not previously been reported to be used in clinical practice. Nevertheless, JAKis should be used with caution and vigilance until studies have clarified the effects of concomitant JAKi and ICI therapies. The implications of JAKi dosing, timing, and length of administration, as well as JAK selectivity and risk factors for VTE or MACE, also need to be elucidated. Clinical trials addressing these issues together with potential advantages of selective JAKi agents over corticosteroids and/or infliximab in the management of irAEs are warranted.

## Data Availability

Data sharing not applicable – no new data generated.
